# The Abductor Digiti Minimi Myocutaneous Island Flap: A Simple Solution for Small Lateral Foot Defects

**DOI:** 10.7759/cureus.97770

**Published:** 2025-11-25

**Authors:** Dmitry Shelepenko, Rui Almeida, Beatriz Garrido, João C Mendes, Luís Teles, Carla Diogo, Miguel Vaz

**Affiliations:** 1 Department of Plastic and Reconstructive Surgery and Burns Unit, Coimbra Local Health Unit, Coimbra, PRT; 2 Department of Orthopedics, Coimbra Local Health Unit, Coimbra, PRT

**Keywords:** abductor digiti minimi, adm, lateral foot reconstruction, myocutaneous flap, soft tissue coverage

## Abstract

The reconstruction of soft-tissue defects of the foot represents a complex surgical challenge, often compounded by unfavorable local conditions, such as active infection, exposure of orthopedic fixation material, and abundant fibrosis of the surrounding tissues, as well as vascular, metabolic, and neuropathic comorbidities. The abductor digiti minimi (ADM) muscle flap is a reliable local option for coverage of lateral foot defects, providing well-vascularized tissue with minimal donor-site morbidity. This study presents a technical variation of the ADM flap that incorporates a vascularized skin island overlying the muscle. The anatomical basis, surgical technique, and two illustrative clinical cases are described.

In both reported cases, reconstruction achieved stable coverage and complete healing without major complications, even in patients with multiple comorbidities and exposed internal fixation material. Inclusion of a skin island enabled immediate coverage, avoiding the need for secondary grafting and resulting in favorable aesthetic and functional integration. This modification of the ADM flap represents a simple, safe, and reproducible technique that expands local reconstructive options for small lateral foot defects. Further studies are needed to confirm its role within the reconstructive algorithm for the foot.

## Introduction

Reconstruction of soft-tissue defects of the foot represents a frequent and complex surgical challenge. These defects, resulting from trauma, chronic infections, pressure ulcers, or postoperative wound complications, often occur in patients with vascular, neuropathic, or metabolic comorbidities and are associated with high morbidity and a significant risk of amputation. Failure to achieve adequate tissue coverage can lead to persistent infection and functional loss of the limb [1]. Several studies have demonstrated that preservation of limb length and integrity directly correlates with improved survival and quality of life, emphasizing the importance of early and effective reconstructive strategies aimed at functional preservation of the foot [2,3].

The abductor digiti minimi (ADM) muscle flap is one of the main local options for coverage of soft-tissue defects of the lateral aspect of the foot, particularly at the level of the ankle, calcaneus, dorsum, and plantar region [1,4,5]. Although its distally based use has been described [6], this flap is most commonly raised in an anterograde fashion as a proximally based muscle flap, supplied by a muscular branch of the lateral plantar artery [5,7]. Its reliable vascularity, ease of harvest, and low donor-site morbidity justify its well-established role in the reconstructive algorithm of the foot [1,7].

This study presents a technical modification of the ADM flap, incorporating a vascularized skin island for the reconstruction of lateral foot defects. The anatomical principles, surgical technique, and two illustrative clinical cases are described to demonstrate its applicability and outcomes.

## Technical report

Anatomy

**Figure 1:**
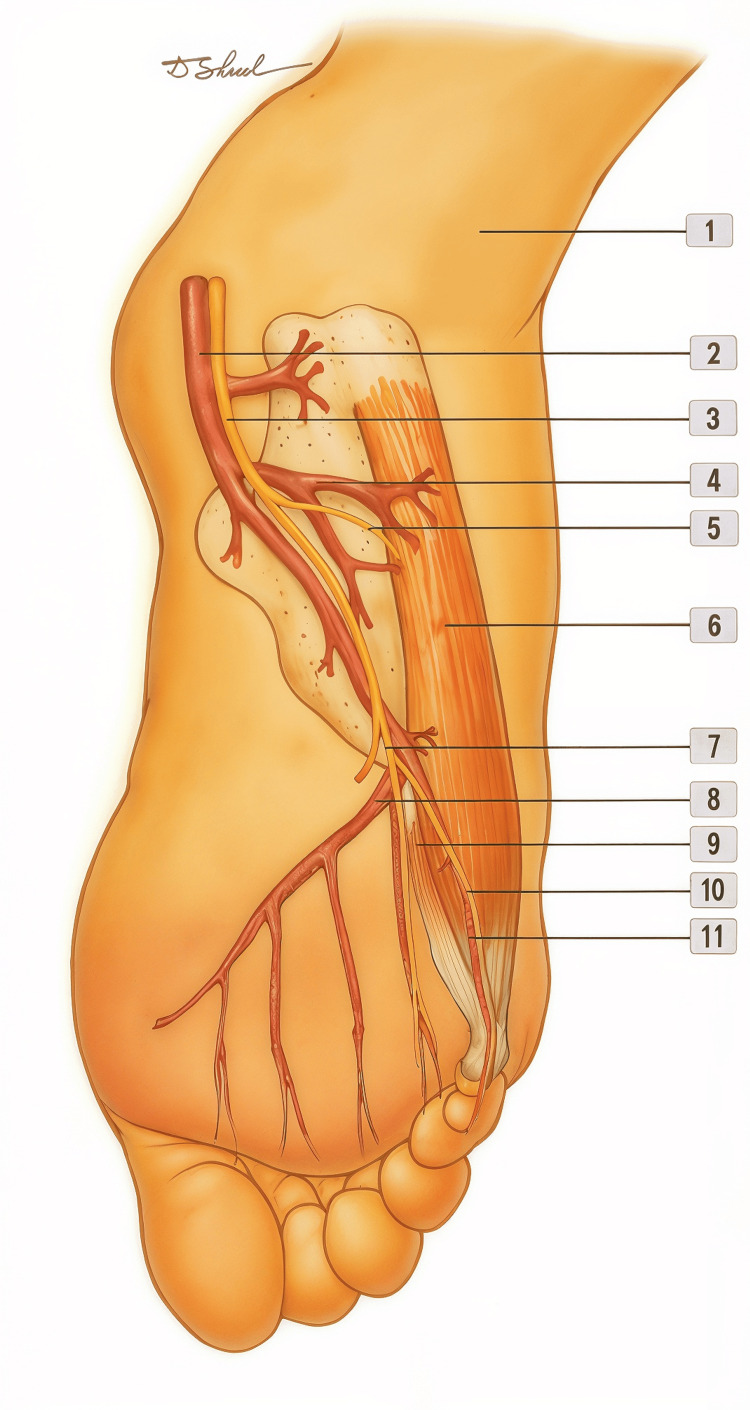
Key anatomical components of the abductor digiti minimi (ADM) flap. The figure presents the ADM muscle and surrounding anatomical structures relevant to the design and elevation of the ADM flap in the plantar view of the foot. 1: Lateral malleolus 2: Lateral plantar artery 3: Lateral plantar nerve 4: ADM artery (dominant pedicle) 5: Motor branch to the ADM muscle (from the lateral plantar nerve) 6: Abductor digiti minimi (ADM) muscle 7: Superficial branch of the lateral plantar nerve 8: Plantar arch (deep plantar arch) 9: Flexor digiti minimi brevis (FDMB) muscle 10: Proper lateral plantar digital nerve to the fifth toe (supplying the lateral side of the fifth toe and innervating the FDMB muscle) 11: Proper lateral plantar digital artery to the fifth toe Image credits: Dmitry Shelepenko.

The ADM is an intrinsic muscle located in the lateral, most superficial layer (first layer) of the plantar aspect of the foot. Its primary function is to abduct the fifth toe. It has a broad posterior origin encompassing both the medial and lateral processes of the calcaneal tuberosity, the intervening bone between them, and the adjacent plantar aponeurosis. From this origin, the muscle belly, broad and thick, gradually tapers to form a tendon near the base of the fifth metatarsal. Its final insertion is on the lateral aspect of the base of the proximal phalanx of the fifth toe, usually in continuity with the tendon of the flexor digiti minimi brevis (FDMB) muscle (Figure 1) [4,5,8,9].

Anatomical variations can be observed in this region. The overlying fascial thickening, corresponding to the calcaneometatarsal band (or the lateral cord of the plantar aponeurosis), may include fibers of the ADM and form an accessory structure known as the abductor metatarsal quinti [9]. In some cases, two distinct muscular portions have been described, separating and rejoining near the base of the fifth metatarsal, creating two separate muscle bellies [5,9].

**Figure 2:**
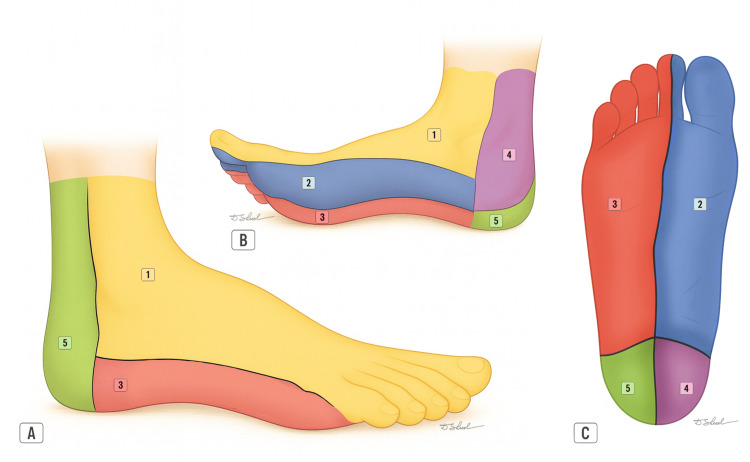
Angiosome perfusion of the foot. The figure illustrates the arterial angiosomes of the foot in lateral (A), medial plantar (B), and plantar (C) views, showing the main vascular territories supplied by: 1: Dorsalis pedis/anterior tibial artery 2: Medial plantar artery 3: Lateral plantar artery 4: Calcaneal branch of the posterior tibial artery 5: Calcaneal branch of the peroneal artery Image credits: Dmitry Shelepenko

The vascular supply and innervation of the ADM arise from the lateral plantar neurovascular bundle, consisting of the lateral plantar artery (a branch of the posterior tibial artery) and the lateral plantar nerve (a branch of the tibial nerve), which courses along the medial border of the muscle within a groove between the ADM and the flexor digitorum brevis (FDB) [8,10,11]. This bundle also provides the cutaneous blood supply to most of the lateral aspect of the foot (Figure 2) and sensory innervation to the distal plantar lateral region [5,12]. Sensory innervation of the proximal plantar lateral and dorsolateral regions of the foot is provided by the terminal branches of the sural nerve [5,13].

The ADM is classified as a Type II muscle according to Mathes SJ and Nahai F, possessing one dominant vascular pedicle and multiple secondary segmental branches [14]. The main pedicle, a consistent branch of the lateral plantar artery, enters the muscle belly near its origin, ensuring predictable and robust perfusion. The secondary pedicles, generally arising from the same artery, supply the middle and distal thirds of the muscle [5,8,10]. Motor branches from the lateral plantar nerve enter the muscle segmentally, primarily in its proximal portion [5,11].

Indications and contraindications

**Figure 3:**
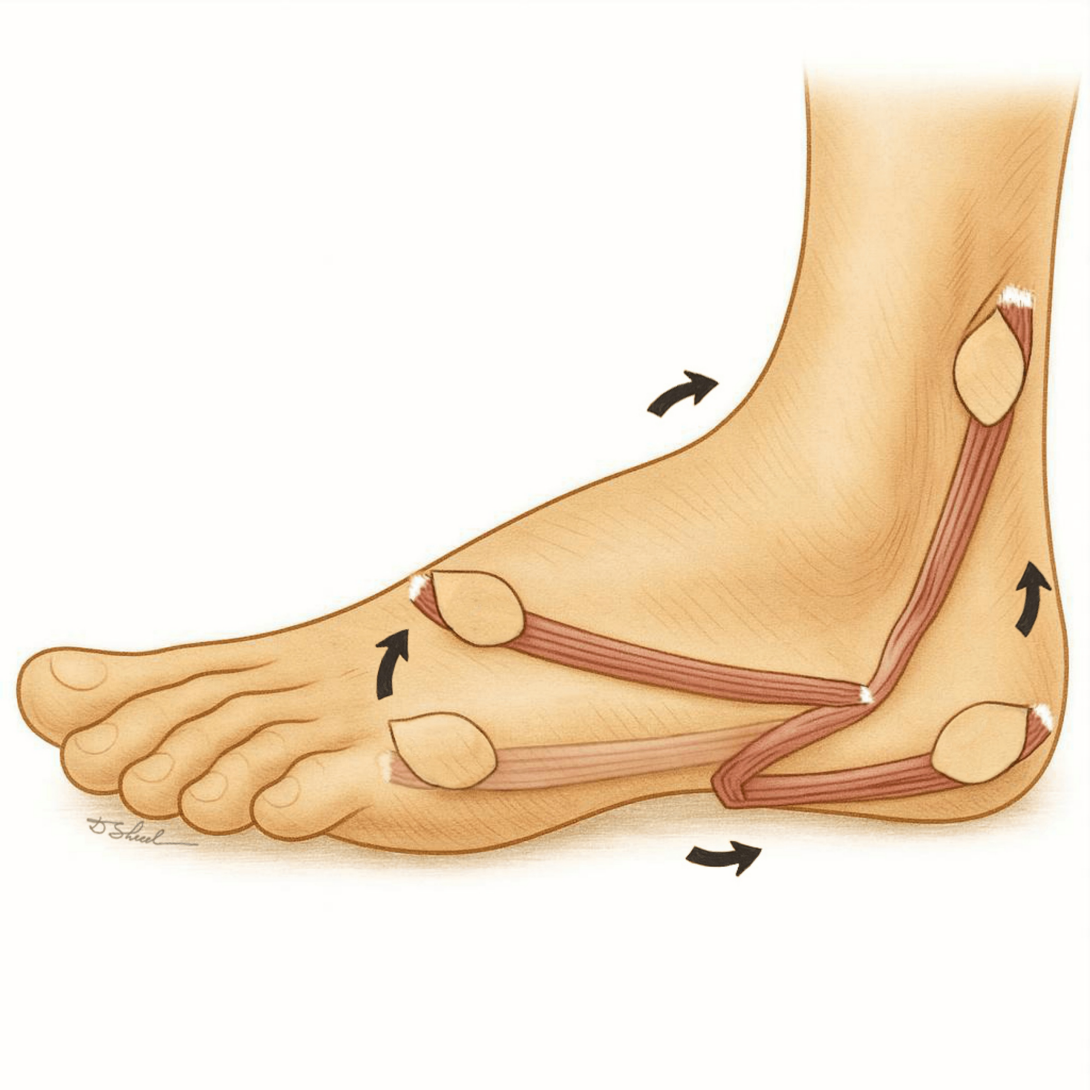
Abductor digiti minimi (ADM) flap mobilization and reach. The elongated form of the muscle allows nearly 360° of rotation, with the maximum pivot point located at the emergence of the ADM artery (dominant pedicle), approximately 2 cm proximal to the styloid process of the fifth metatarsal bone. Image credits: Dmitry Shelepenko

The elongated shape of the ADM muscle and its nearly 360° arc of rotation provide excellent versatility, allowing coverage of small defects located on the lateral, dorsal, or calcaneal regions of the foot (Figure 3) [1,11]. The flap is particularly useful when stable coverage with well-vascularized tissue is required in areas with unfavorable local conditions, namely in cases of exposed bone, tendon, or fixation hardware, where skin grafts are not viable and fasciocutaneous flaps may be insufficient. Accordingly, the ADM flap can be employed for defects of multiple etiologies, including traumatic wounds, chronic ulcers, postoperative infections, and localized osteomyelitis [1,5].

Relative contraindications include extensive defects exceeding the flap’s reach, proximal vascular compromise of the lateral plantar artery (secondary to trauma or prior surgery), and cases with significant damage to the lateral aspect of the foot, which may affect the integrity of the muscle or, in musculocutaneous island designs, the overlying skin. The flap should also be used with caution in patients with advanced peripheral arterial disease or uncontrolled infection, in whom flap viability and functional integration may be compromised [5,7,8]. Preoperative assessment of the vascular status of the foot is therefore essential, with particular attention to the posterior tibial artery and its lateral plantar branch, which must be patent to ensure adequate perfusion of the flap. A thorough Doppler examination should be performed to evaluate both antegrade and retrograde components of the pedal circulation. In cases of doubtful vascular status, angiography is recommended to confirm vessel patency and minimize the risk of ischemic complications [5,7,11]. Nevertheless, in selected patients with compromised vascularity who are not candidates for revascularization, transfer of muscle tissue may represent the only effective means of enhancing local oxygenation and facilitating the delivery of immune cells and systemically administered antibiotics to the affected area, thereby promoting infection control and wound healing [1].

Surgical technique

**Figure 4:**
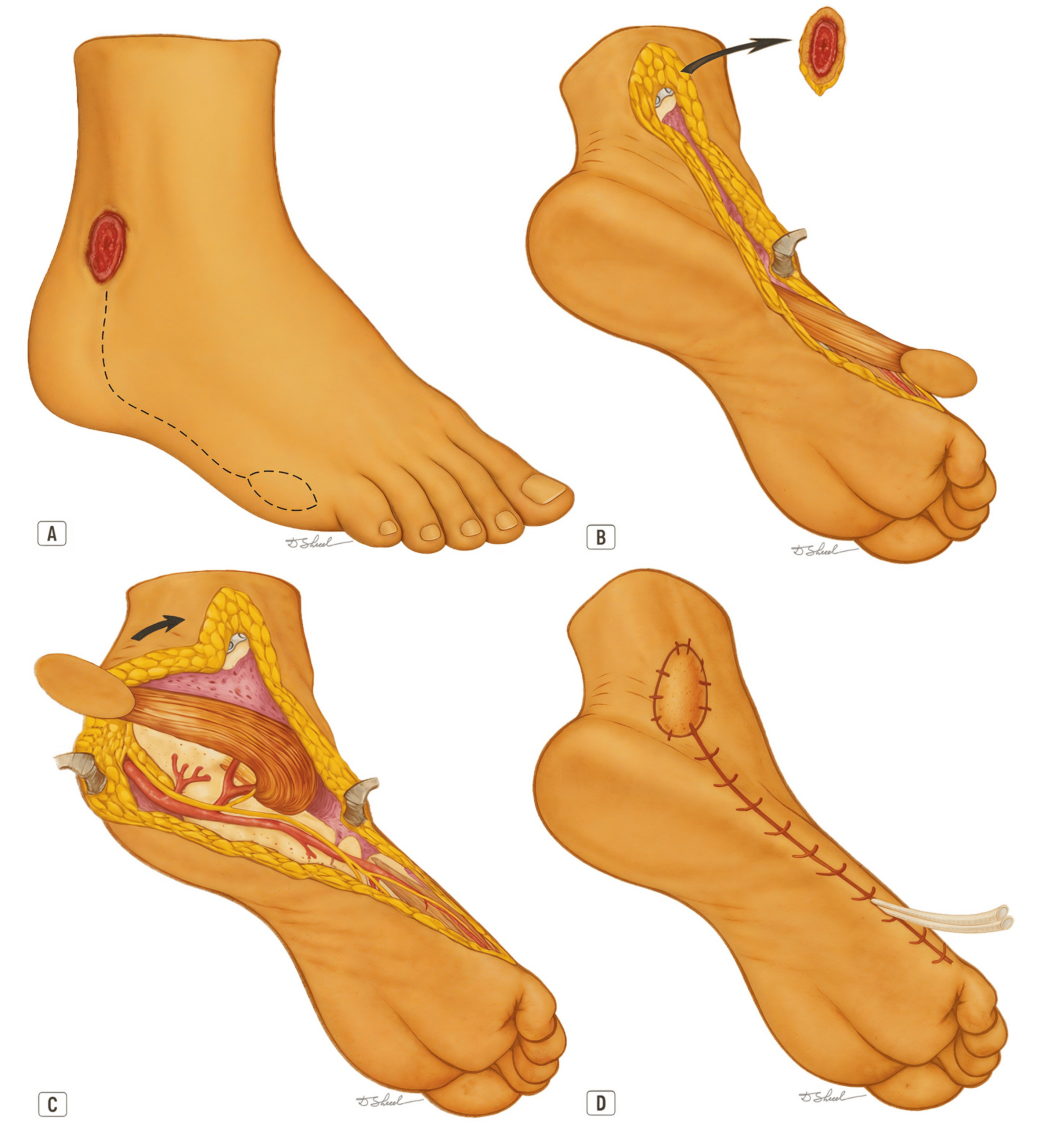
Stepwise surgical technique of the abductor digiti minimi (ADM) island flap. The figure presents a stepwise surgical overview of the abductor digiti minimi (ADM) island flap for coverage of a lateral malleolus defect (ulcer). (A) ADM island flap markings, with the island positioned just proximal to the base of the fifth toe and the incision line following the lateral border of the foot, extending toward the defect; (B) Defect excision and flap elevation; (C) Flap rotation to the defect with preservation of the lateral neurovascular bundle, the dominant artery to the flap, and the flexor digiti minimi brevis (FDMB) muscle (the flexor digitorum brevis (FDB) muscle, located superficial to the lateral plantar artery and nerve, is not shown to allow better visualization of these structures); (D) Skin closure after flap transposition, with multitubular drains placed beneath the suture line. Image credits: Dmitry Shelepenko

With the patient in the supine position and the lower limb internally rotated, a pneumatic tourniquet is placed on the thigh and the extremity is prepared in the usual sterile fashion. After meticulous debridement of the recipient bed and confirmation of the viability of surrounding tissues, flap planning is performed, including the design of the skin island and marking of the rotation (pivot) point. The skin island is designed in an elliptical shape, with a maximum width of approximately 2 cm to allow primary closure of the donor site and avoid skin compromise. It is centered over the middle or distal portion of the ADM muscle, along the lateral border of the foot, within the angiosome territory of the lateral plantar artery (Figure 4).

After flap design and confirmation of landmarks, the limb is elevated and the tourniquet inflated. A straight or lazy-S incision is made along the lateral border of the fifth metatarsal, extending from the calcaneal region to the base of the proximal phalanx of the fifth toe. In most cases, the incision is prolonged proximally toward the defect to be reconstructed. At the level of the skin island, the initial incision may pass either superior or inferior to the island, allowing later adjustment of the design as needed. This approach exposes the underlying muscle belly and its distal tendinous insertions, typically located on the lateral aspect of the base of the proximal phalanx of the fifth toe and, occasionally, also at the base of the fifth metatarsal. When this anatomical variation is encountered and the ADM presents with two distinct muscle bellies, the distal belly may easily be mistaken for the adjacent FDMB muscle, which lies medial to the ADM (Figure 1).

Once the medial and lateral borders of the muscle have been identified, the skin-island design is readjusted and the incision completed, ensuring that the island remains centered and in full contact with the ADM. From this stage onward, the dissection follows the same sequence as for the pure muscle flap.

Dissection proceeds in the intermuscular plane from distal to proximal along the medial border of the muscle, where the lateral plantar neurovascular bundle is identified and preserved. The muscle is carefully released from its distal tendinous insertions, the adjacent fascial structures, the connections to the FDMB, and finally from its bony attachment to the base of the fifth metatarsal. Small segmental pedicles arising from the neurovascular bundle along the middle and distal thirds of the muscle are ligated and divided to allow greater flap mobility [1,5,8,10]. It is important to emphasize that the lateral plantar neurovascular bundle, as well as its terminal arterial and neural branches to the fifth toe, should not be elevated together with the flap [13].

Dissection ends approximately 2 cm proximal to the styloid process of the fifth metatarsal, where the main vascular pedicle enters the muscle belly near its origin. This vessel is identified and protected, avoiding excessive skeletonization [4,8]. If additional mobility is required, the muscle can be detached from its broad calcaneal origin, leaving it attached only by its main vascular pedicle [5,9].

The tourniquet is then deflated, and flap perfusion is verified. After hemostasis, the flap is rotated and positioned over the defect, ensuring that there is no tension, torsion, or compression of the vascular bundle. The flap is secured in place with absorbable subcutaneous sutures and interrupted skin stitches. The donor site is closed primarily in the same manner, and multitubular drains are typically placed to prevent hematoma formation [8,10].

In cases where the muscle is transferred without a skin island, or when the skin paddle is insufficient to achieve complete coverage, the defect can be supplemented with a split-thickness skin graft, applied either immediately or in a delayed fashion according to local conditions [5].

Throughout the procedure, meticulous dissection under loupe magnification, the use of bipolar cautery, and delicate instrumentation are recommended. Intraoperative Doppler assessment can be used to confirm flow within the main pedicle before and after flap rotation [1,5,15].

Case 1

**Figure 5:**
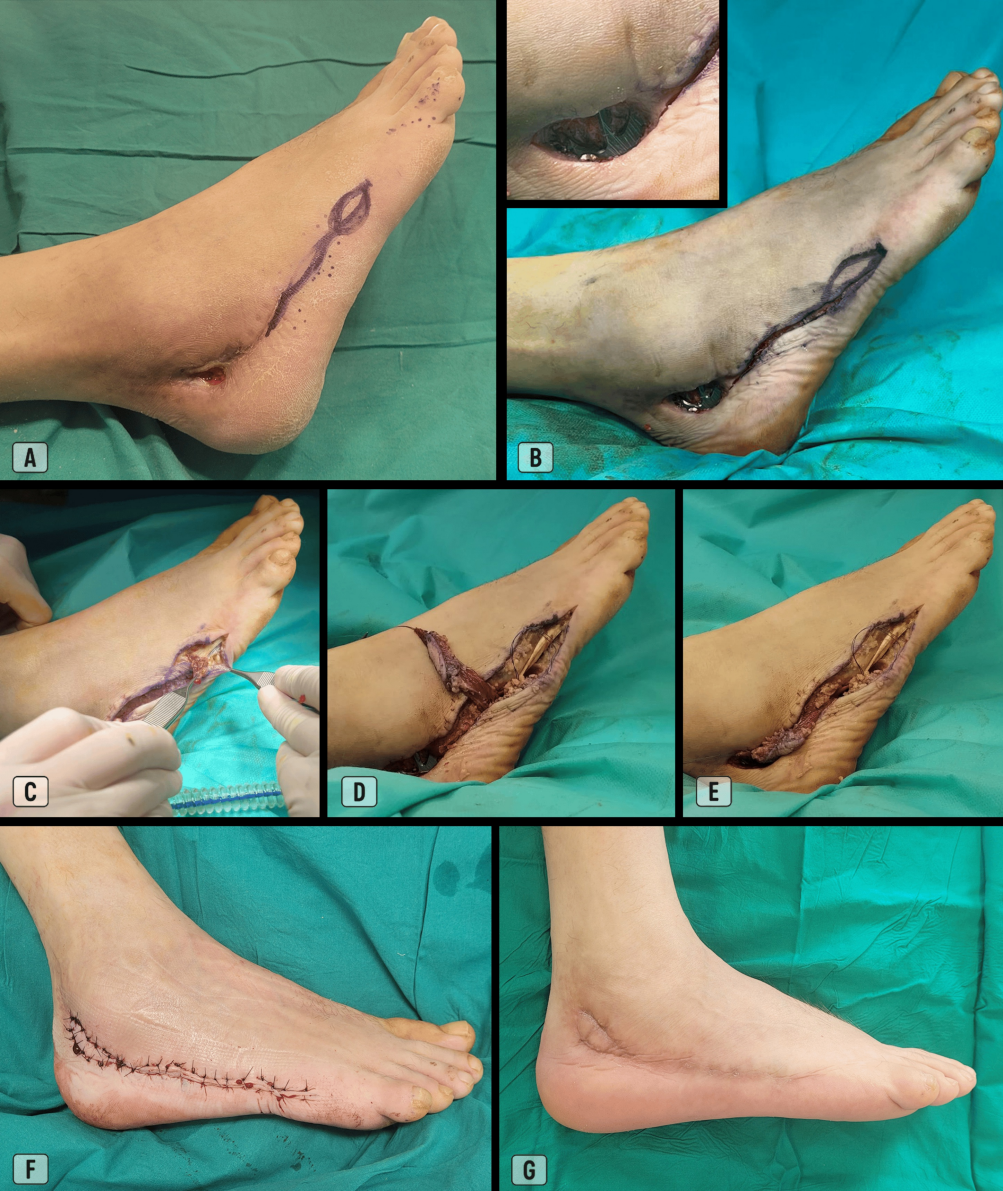
Case 1. (A) Pre-operative markings of the incision and cutaneous island; (B) Defect after debridement and incision along the lateral border of the foot (inset: close-up view showing exposure of the calcaneal plate); (C) Elevation of the ADM flap with cutaneous island, from distal to proximal; (D) Completion of flap elevation (pivot point at the base of the fifth metatarsal); (E) Flap reach to the defect site; (F) Flap adaptation and skin closure (combination of Allgöwer-Donati and horizontal mattress sutures; Ethilon® 4-0); (G) Result 6 months after surgery. ADM: Abductor digiti minimi.

A 44-year-old male patient with diabetes mellitus, hypertension, active smoking habits, and a history of chronic alcoholic pancreatitis presented with a comminuted fracture of the right calcaneus, which had been treated surgically by open reduction, bone grafting, and internal fixation using an anatomical plate and screws. The postoperative course was complicated by wound dehiscence posterior to the lateral malleolus, which subsequently evolved into an ulcerated lesion measuring approximately 3 × 2 cm, with exposure of the fixation hardware. Two months after the initial surgery, the patient underwent surgical debridement and coverage of the defect with an ADM flap incorporating a skin island (Figure 5). The procedure was uneventful, and placement of a drain was not required. Sutures were removed on the 20th postoperative day.

Although the flap remained fully viable, healing was delayed due to the formation of a small fistulous tract between the anterior suture margin and the deeper tissues, where the fixation hardware was located. Complete spontaneous closure of the fistula occurred over the following weeks with antibiotic therapy and standard wound care, mostly managed on an outpatient basis, without requiring removal of the plate and screws. At the time of the follow-up assessment, approximately 6 months postoperatively, the scar was fully closed with stable maturation.

Case 2

**Figure 6:**
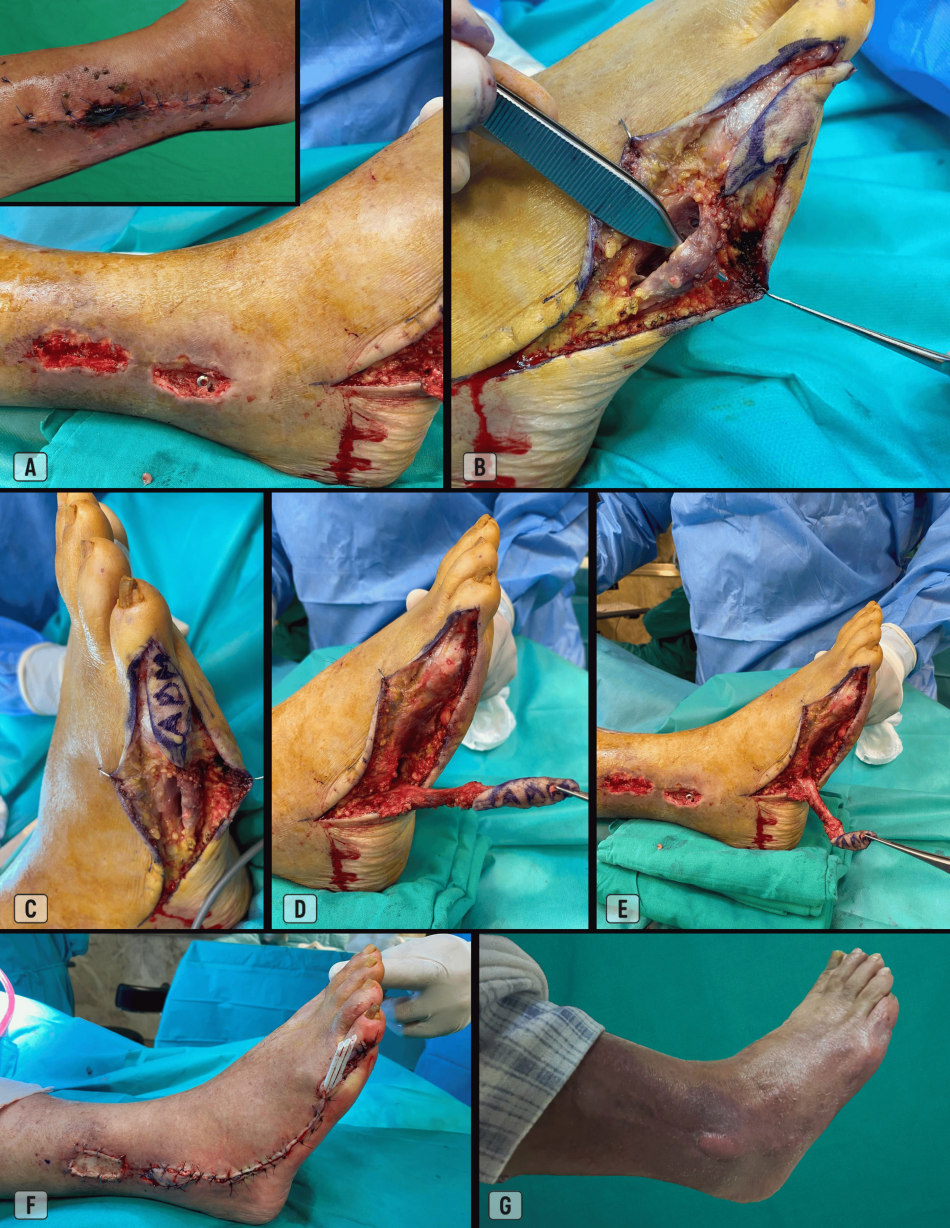
Case 2. (A) Defects after debridement of necrotic plates, with exposure of the peroneal plate in the lower lesion (inset: pre-operative view, 3 weeks before surgery); (B, C) ADM flap elevation, the cutaneous island was extended 2 cm distally into the lateral dorsal skin of the fifth toe to achieve adequate reach; (D) Completion of flap elevation (pivot point 2 cm proximal to the styloid process of the fifth metatarsal); (E) Rotation of the flap into the defect site; (F) Flap adaptation and skin closure (combination of simple and vertical mattress sutures; Ethilon® 4-0). The upper defect was covered with a split-thickness skin graft; (G) Result 4 months after surgery.

An 82-year-old male patient, previously independent in daily activities and with a history of severe ischemic heart disease, presented after a fall with distal-third fractures of the bones of the right leg (Weber type C). He underwent internal fixation with plates and screws of the tibia and fibula, later complicated by wound dehiscence and necrosis of the cutaneous flaps over the lateral malleolus, leading to exposure of the fixation hardware. The patient was admitted for reconstruction of the defect two months after the initial dehiscence, once the infection had been controlled. After debridement of devitalized tissue, a defect measuring 1.5 × 4 cm along its greatest vertical axis was identified. Coverage was achieved using an ADM flap with a skin island. The surgical procedure is illustrated in Figure 6.

The postoperative course was largely uneventful, aside from mild epidermolysis and delayed healing at the distal margin of the skin island, likely related to poor compliance with postoperative positioning, frequent agitation, and the distal, off-center placement of the skin island over the underlying muscle. The multitubular drain was removed on the second postoperative day, and the skin sutures on day 17. Ambulation was resumed after three weeks, with complete healing achieved by 4 months of follow-up.

## Discussion

The ADM muscle flap has become a well-established option within the reconstructive armamentarium of the foot, widely recognized for its reliability and technical simplicity [8]. Since its original description by Ger R in the 1970s and 1980s [9,16-18], it has been used primarily as a pure muscle flap, either alone or in combination with other intrinsic muscle flaps of the foot [7,15]. This classic configuration has proven to be an effective solution for the reconstruction of various lateral-foot defects, combining consistent results with low donor-site morbidity. The flap is particularly valuable in patients with significant comorbidities, local infection, or abundant surrounding scar tissue, situations in which local fasciocutaneous flaps are limited and microsurgical options carry greater risk [8,10].

A recent systematic review published by Gkotsoulias E and Kuten D (2025) on the use of intrinsic muscle flaps for the treatment of calcaneal osteomyelitis, including 14 studies and 102 patients treated with the ADM flap, reported a 100% healing rate, with only isolated cases of partial skin-graft loss [7].

To the best of our knowledge, the use of the ADM flap incorporating a vascularized skin island has not previously been described in the literature. Although no specific reason for this omission has been identified, historically there has been a general tendency to avoid musculocutaneous flaps in the foot, possibly due to the inherent bulk of such flaps, the considerable variability in the location of muscle perforators in the lower extremity, the risk of unacceptable donor-site defects, and the consistently good results achieved with both classical muscle-based and more recent perforator-based flap techniques [9,11,19]. Consequently, most published clinical series describe the ADM exclusively as a muscle flap with immediate or delayed skin grafting [7].

In our experience, the inclusion of a small skin island centered over the middle or distal portion of the muscle proved to be a useful and effective modification. At first glance, one might assume that ligation of the direct perforators and distal minor pedicles to the ADM would compromise the vascularity of the island; however, perfusion is adequately maintained through musculocutaneous perforators that, although divided at their emergence from the lateral plantar artery, maintain perfusion through an extensive intramuscular choke-vessel network supplied by the proximal dominant pedicle [20].

The main advantage of this modification is the ability to achieve immediate cutaneous coverage without the need for skin grafting, thereby eliminating the risk of graft loss due to infection or hematoma and potentially reducing both the number of operative procedures and the time to definitive reconstruction. An additional benefit lies in the fact that the flap provides a coverage surface with thickness and texture similar to the surrounding skin, offering greater resistance to trauma and improved tolerance to early weight bearing [11,13].

Among the limitations, the relatively small coverage area remains the most significant constraint of this technique. The presence of healthy overlying skin is also essential to ensure viability of the island. Isolated episodes of superficial epidermolysis may occur, possibly related to limited venous drainage, although no cases of flap failure or skin-island necrosis were observed in our series. Even in Case 2 (Figure 6), where approximately 70% of the distal portion of the skin island was not directly centered over the muscle, the flap survived without significant complications.

Another limitation is the absence of sensibility in the skin island, an important consideration in calcaneal reconstruction, where plantar sensitivity is critical for functional gait. In such situations, alternative or combined techniques, such as association of the ADM flap with the lateral calcaneal artery flap (which includes the sural nerve), as described by Al-Qattan MM [13], may represent a more suitable option.

From a technical standpoint, although flap dissection is relatively straightforward, precise knowledge of the local anatomy and its variations, particularly the distal tendinous insertions, the course of the lateral plantar neurovascular bundle, and the relationships with the FDB and FDMB muscles, is essential to avoid intraoperative confusion. Unpredictable circumstances may arise: the defect may prove larger than anticipated, or the flap may not reach the desired area. Therefore, the surgeon should always have an intraoperative backup plan available, including the possibility of conversion to a free flap if necessary [1].

## Conclusions

The cases presented demonstrate the reliability of this technical variation, achieving satisfactory functional and aesthetic outcomes even in patients with multiple comorbidities and the presence of orthopedic fixation material within the wound bed. The technique preserves the simplicity and predictability of the classic muscle flap while adding the advantage of immediate skin coverage, eliminating the need for grafting and providing excellent morphological integration with the surrounding skin.

Although our experience remains limited, the results obtained suggest that inclusion of a skin island in the ADM muscle flap represents a safe, reproducible, and potentially advantageous modification. Further studies with larger cohorts and longer follow-up are needed to better define the role of this technique within the reconstructive algorithm of the lateral foot, as well as to delineate its precise indications and limitations.
